# The Effect of Oral Magnesium Supplement on Postoperative Pain Following Mandibular Third Molar Surgery: A Split-Mouth Randomized Placebo-Controlled Trial

**DOI:** 10.1155/prm/7157801

**Published:** 2025-01-23

**Authors:** Sutthipat Nimkulrat, Phichayut Phinyo, Warit Powcharoen

**Affiliations:** ^1^Graduate School, Faculty of Dentistry, Chiang Mai University, Chiang Mai 50200, Thailand; ^2^Department of Biomedical Informatics and Clinical Epidemiology (BioCE), Faculty of Medicine, Chiang Mai University, Chiang Mai 50200, Thailand; ^3^Center for Clinical Epidemiology and Clinical Statistics, Faculty of Medicine, Chiang Mai University, Chiang Mai 50200, Thailand; ^4^Department of Oral and Maxillofacial Surgery, Faculty of Dentistry, Chiang Mai University, Chiang Mai 50200, Thailand

## Abstract

**Objective:** This study aimed to evaluate the analgesic efficacy of oral magnesium supplements, administered as an analgesic adjuvant to ibuprofen, on acute postoperative pain within 72 h following mandibular third molar (MTM) surgery.

**Materials and Methods:** This triple-blind, placebo-controlled, split-mouth randomized study was conducted among 25 patients (50 MTMs), who intended to remove both MTMs. All patients underwent two surgeries separated by an interval of at least 4 weeks. For each surgery period, patients were randomly assigned with either receiving NSAIDs plus oral magnesium supplement (25 MTMs) or NSAIDs plus placebo (25 MTMs) for three days after surgery. The postoperative pain intensity at rest and movement were primarily evaluated at 24 h, postoperatively. Participants were also asked to record pain intensity at 6, 48, and 72 h, postoperatively, rescue analgesic consumption, time to first rescue analgesic, and magnesium-related adverse events.

**Results:** The combination of ibuprofen plus oral magnesium supplement significantly decreased pain intensity at rest 24 h, postoperatively, compared to placebo (estimated mean difference −15.08; 95%CI −29.01 to −1.14). However, the pain intensity at rest and movement were similar between groups at other time points. There was no significant difference among groups in terms of rescue analgesic consumption and time to first rescue analgesic. No magnesium-related adverse event was observed.

**Conclusion:** The addition of oral magnesium supplement as an analgesic adjuvant to NSAIDs significantly decreased pain intensity at rest 24 h following MTM surgery. Nevertheless, this result might not provide clinically relevant benefits for pain control following MTM surgery.

**Trial Registration:** ClinicalTrials.gov identifier: TCTR20221003004

## 1. Introduction

Over the decade, postoperative pain following third molar surgery has been undesirable sequelae for both dentist and patient. Poorly controlled postoperative pain can lead to increased morbidity and decreased physical function [1]. To enhance recovery, numerous pharmacological and nonpharmacological interventions have been developed to alleviate this postoperative sequelae and improve patient's quality of life. In addition, recent studies have explored the potential of newer pharmacological agents, such as esketamine [2], tramadol [3], diclofenac [4], and lornoxicam [5], to further improve postoperative pain control following third molar surgery. The advancements in pharmacological approaches have also focused on combining different classes of medications to achieve synergistic effects. Multimodal pain management protocols for third molar surgery, which integrate various pharmacological agents, have become interested for dentists in providing superior pain relief while minimizing their associated side effects.

Magnesium was introduced to postoperative application and resurfaced in the era of enhanced recovery after surgery protocol and multimodal analgesic plans. Magnesium is a physiological and pharmacological blocker of N-methyl-D-aspartate (NMDA) receptors in the neuronal tissue [6]. In daily practice, magnesium is also available as an over-the-counter dietary supplement without prescription. This oral magnesium supplement is a safe and low-cost alternative for varied diseases or condition [7], but the analgesic effect of this type of magnesium remains unclear. Administration of magnesium, especially intravenous route, has been extensively studied on postoperative pain management [8], whereas oral magnesium supplement has been rarely investigated.

Previous studies among dental treatment or oral surgery, focusing on the administration of magnesium and postoperative pain, are limited to the studies by authors in [9, 10]. At present, there are few evidence supporting the use of oral magnesium supplement in dentistry, also third molar surgery. Therefore, the aim of this study is to evaluate the analgesic efficacy of oral magnesium supplement (500 mg) that was orally administered as an analgesic adjuvant on postoperative pain intensity within 72 h following mandibular third molar (MTM) surgery.

## 2. Materials and Methods

### 2.1. Participants

Participants were patients who were scheduled for bilateral MTM surgery at Oral and Maxillofacial Surgery Clinic, Faculty of Dentistry, Chiang Mai University. Ethics approval was obtained from the Ethics Committee of the Faculty of Dentistry, Chiang Mai University (protocol no.063/2022). The written informed consent was obtained from all participants.

The inclusion criteria included healthy patients aged 18 years or above. Both MTMs required the bone removal and tooth sectioning to allow the tooth to be extracted. The exclusion criteria included pregnant or breast-feeding women, those with a history of systemic diseases or conditions contraindicated to magnesium administration, those with ongoing orofacial pain on the day of surgery, those who had taken any medication or supplement within 1 week prior to surgery, and those with third molar–associated infection or pathology. The participants who failed to undergo both MTM surgeries took any medication or supplement other than those in this study or developed postoperative complications were withdrawn from the study.

### 2.2. Study Protocol

The study was a triple-blinded, placebo-controlled, randomized split-mouth design. A random sequence was generated via an online website (https://www.sealedenvelope.com) by independent person to assign eligible patients (1:1 allocation ratio) into two sequences, consisting of magnesium–placebo (Mg–placebo) or placebo–magnesium (placebo–Mg). The generated randomization was stratified by preoperative anxiety level (high or low anxiety) with random permuted blocks of four and eight. The preoperative anxiety level of the participants was measured while they were waiting in the reception area by using the Thai version of the Modified Dental Anxiety Scale (MDAS) [11]. Allocation concealment was done with sealed opaque envelopes, which were opened by blind surgeons, immediately prior to the surgical procedure.

Magnesium supplements and placebo were prepared with identical appearance and kept in the amber pill bag by independent person. Each bag contained four capsules of magnesium supplement or placebo, which blinded dentists and participants. For magnesium arm, participants received an amber pill bag containing 500 mg magnesium supplement (Life Extension, Thailand) after randomization and took one capsule prior to surgery. Postoperatively, participants received 400 mg ibuprofen three times a day and 500 mg magnesium supplement once a day at the same time as the first dose for 3 days. For the placebo arm, participants received placebo instead of magnesium supplement which was scheduled similar to magnesium arm.

To qualify, the dentists who were responsible for third molar surgery were required to provide proof of standard MTM surgery with supervision. Specifically, they needed to have completed a minimum of 20 surgeries, consisting of 10 on each side of the MTMs. The operative time for each surgery should have been less than 60 min. In addition, the complication rate (including nerve injury, alveolar osteitis, soft tissue injury, fracture of the lingual plate, injury to the second molar, or excessive bleeding) had to be less than 5%. On the day of surgery, participants were evaluated for preoperative dental anxiety and pain catastrophizing. A history, clinical, and radiographic examination were undertaken. The first surgery randomly operated on only one side of MTMs. Participants underwent surgery with the standard operating procedure under local anesthesia (with two cartridges of 4% articaine with 1:100,000 epinephrine at the beginning). Additional use of local anesthetics during surgery and the total operation time were recorded. Participants were prescribed amoxicillin or clindamycin for 7 days, and 500 mg paracetamol as rescue analgesic. The total number of rescue analgesics used was also recorded. A wash-out period of at least 1 month was required between two surgeries to eliminate the crossover effect of previously used medications and possible surgical site infection. Consolidate Standards of Reporting Trials (CONSORT) guidelines [12] were followed reporting the results of the study.

### 2.3. Outcome Measurement

The primary outcome was postoperative pain intensity, measured by Heft–Parker visual analog scale (HP-VAS) [13], at 24 h after surgery. Both pain at rest and movement (during maximal mouth opening) were recorded. Moreover, participants were asked to record the secondary outcomes which were postoperative pain intensity at 6, 48, and 72 h after surgery, the time taken for the first rescue analgesic and pain intensity at the time of first rescue analgesic consumption. The occurrence of magnesium-related adverse events within 1 week after surgery was observed.

### 2.4. Statistical Analysis

The sample size calculation was based on a two-sample paired-means test and the result of the previous study [9]. The total number of 25 participants (50 MTMs) was required to detect a difference of visual analog scale of 2.05 (with 80% power and 0.05 level of significance), assuming standard deviation of 1.54 and 2.86; and the correlation coefficient of −0.32 between groups.

To account for the correlated nature of data, statistical methods carefully considered both time and cluster effects. Intention-to-treat analysis was used. The multilevel linear mixed-effects modeling was employed to analyze the primary outcome. The fixed effect included the full interaction of treatment and period effects with time. Three-level data structures for the random parts were defined, where patients were at Level 3, teeth were at Level 2, and each repeated observation was at Level 1. The model estimated the mean difference between groups with the corresponding 95% confidence interval (CI). All data were analyzed using Stata/BE version 18 (StataCorp LLC, Texas, USA).

## 3. Results

### 3.1. Baseline Characteristics

**Figure 1:**
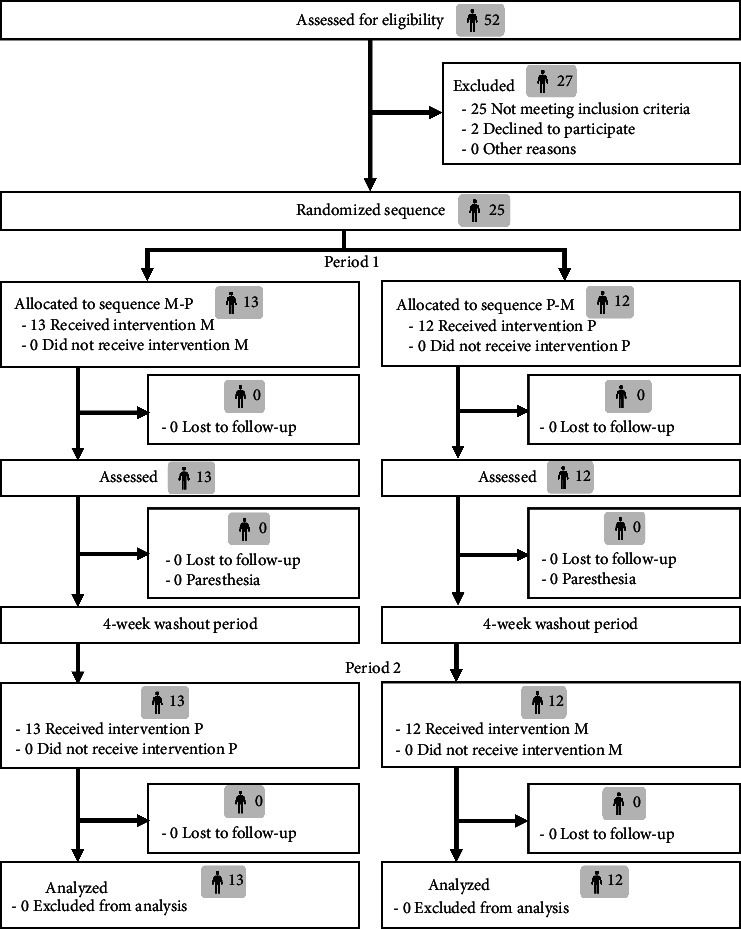
CONSORT diagram of the study.

Twenty-five participants (50 MTMs) were screened and included in this study. All participants were randomly assigned to sequence Mg–placebo (*n* = 26) or placebo–Mg (*n* = 24). There were no losses and exclusions after randomization as shown in the CONSORT participant flow diagram (Figure 1). The CONSORT 2010 checklist of information to include when reporting a randomized trial was presented in Appendix Table A1. Baseline demographic and characteristics of participants, regarding sequence and total, are presented in Table 1. At baseline, the treatment sequences were generally comparable.

### 3.2. Efficacy and Safety

**Figure 2:**
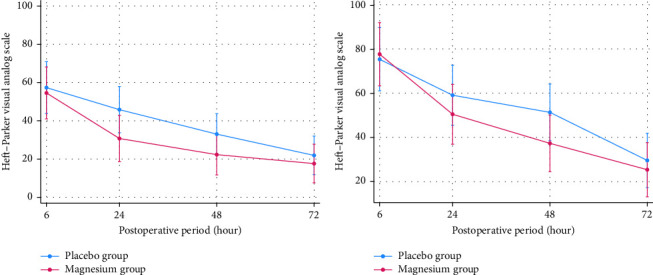
(a) Marginal prediction plots visualize trend of postoperative pain at rest 6–72 h. (b) Marginal prediction plots visualize trend of postoperative pain at movement 6–72 h.

At 24 h after surgery, oral magnesium supplement was significantly better than placebo for controlling pain at rest (mean ± SD HP-VAS, 30.80 ± 29.95 vs. 46.04 ± 31.19; *p*=0.044) with the estimated treatment difference of −15.08 (95%CI; −29.01 to −1.14). There were not statistically significant differences of pain at rest and movement between groups across other time points (Table 2). The marginal prediction plots visualized the downtrends of postoperative pain intensity at rest and movement on both oral magnesium supplement and placebo group (Figure 2). The trend of both postoperative pain intensity at rest and movement in the oral magnesium supplement group was obviously lesser than the placebo group for 24–48 h, postoperatively.

The number of participants who took rescue analgesic was 10 (40%) in the magnesium group and 14 (56%) in the placebo group. Among those who consumed rescue analgesic, both groups showed similar total number of consumed rescue analgesic, time to first analgesic consumption, and pain intensity at the time of first rescue analgesic consumption within 72 h following surgery. None of the participants developed magnesium-related adverse events within 1 week after surgery. Secondary outcomes are summarized in Table 3.

## 4. Discussion

Postoperative pain usually worsens during the first 3 days following MTM surgery, impairing the patient's daily activities and quality of life. Pharmacological management is essential for enhancing the short-term quality of life of patients who are undergoing third molar surgery. To improve patient comfort and expedite the return to normal activities, it is imperative to implement effective pain control and minimize postoperative complications, including edema and trismus. Multimodal pain strategies have been investigated to alleviate this undesirable pain, but the best strategy remains inconclusive. The key finding of this randomized controlled study is that oral magnesium supplement significantly decreased pain at rest at 24 h following MTM surgery in setting that ibuprofen was primary analgesic. Even though the difference was not significant, there were less participants in the oral magnesium supplement group who took rescue analgesic (paracetamol). This may suggest better pain control from adding oral magnesium supplement to ibuprofen consumption.

The analgesic effect of magnesium relates to an NMDA receptor antagonist. One possible mechanism is the binding of magnesium to its specific location on voltage-dependent ion channels such as NMDA receptor, which inhibits calcium influx and prevents central sensitization from peripheral nociceptive stimulation [14]. Previous studies raised the benefit of postoperative magnesium administration (intravenous, intrathecal and/or epidural, and intra-articular) that significantly decreased postoperative pain and improved outcomes related to pain after surgery [8]. In fact, magnesium is also available as an oral dietary supplement which is safe, inexpensive, and available over the counter. However, very few studies focused on the analgesic efficacy of oral magnesium supplements in certain conditions that is fibromyalgia [15] and chronic pain [7].

The commonly used magnesium pharmaceutical form in postoperative pain management is magnesium sulfate, which the optimal dose for analgesic effect and decreasing consumption of additional analgesics are still a controversy [16, 17]. However, the use of magnesium sulfate appears to be not clinically feasible in dental settings, regarding intravenous administration. Recently, oral magnesium supplements have become interesting for pain alleviation as an adjunctive analgesic. The significant benefits of oral magnesium supplement or when combined with other analgesics in the reduction of pain were reported in previous clinical studies [18–21], but the contradicted results were also reported [22–24]. The pharmacological efficacy of magnesium is significantly influenced by its bioavailability [25]. The different generations of magnesium salt exhibit a wide range of bioavailability, which magnesium oxide, citrate, and chloride showed a better bioavailability, compared with other salts [17]. The oral magnesium supplement used in this study consists of magnesium oxide, citrate, and succinate that lead to good bioavailability and possibly better onset of analgesic effects. Furthermore, magnesium will exhibit greater relative bioavailability when ingested in multiple low doses over the course of the day, as opposed to single high-dose ingestion [16]. This might support the finding that magnesium significantly relieves pain at the 24th hour following surgery, not earlier. Because none of the previous studies suggested the best regimen of pain alleviation for oral magnesium supplement, this study proposed the once daily dose as recommended by a product which is simple and clinically feasible.

Previously, only one clinical trial investigated the efficacy of oral magnesium on postoperative pain following MTM surgery [9]. Jerkovic et al. revealed the analgesic effect of magnesium citrate in tablets (400 mg once daily for 3 days) and lozenge (100 mg every 6 h for 3 days) form as primary analgesic for 3 days following MTM surgery. The use of magnesium citrate tablets also significantly decreased recue analgesics (ibuprofen) consumption on the first postoperative day. In contrast to this study, the use of oral magnesium supplement only resulted in a significant reduction of pain at rest on the first postoperative day and failed to decrease recue analgesics (paracetamol) consumption and time to first rescue analgesic consumption. To clarify, this study identified the effect of oral magnesium as an analgesic adjuvant combined with NSAIDs, rather than the effect as primary analgesic similar to the previous study [9]. Moreover, there was a different composition among pharmaceutical forms used between these two studies.

The administration of oral magnesium supplements is perhaps followed by some adverse events, such as diarrhea or other gastrointestinal conditions [26], and risk of high magnesium levels. The U.S. Food and Drug Administration recommended the daily dose of 420 mg for adults and children aged 4 years and older [27]. Most previous studies administered oral magnesium supplements that comply with FDA recommendations, with exception of few clinical trials [26, 28, 29]. However, none of magnesium-related adverse events was observed in this study.

Even though NSAIDs has become primary analgesic for pain management following MTM surgery, poorly controlled postoperative pain has been reported in 16%–20% of the patients [30, 31]. The statistically significant result of this study suggested the combination of NSAIDs and oral magnesium supplements as alternative medication, for controlling undesirable pain at rest on the first postoperative day following MTM surgery. However, this effect size of pain reduction should be carefully interpreted for meaningful clinical significance. Because the upper limit of 95% CI of this effect size was below expected minimal clinically important difference, this result of pain reduction was definitely not clinically significant [32].

Strengths of this study include blinding of study treatments and a split mouth or cross-over design that patients were assigned to both magnesium and placebo in similar health states. Because each individual patient serves as his or her own control, a substantial amount of intersubject variation is eliminated. Moreover, the 4 week washout period and random assignment minimized possible carryover and sequence effects. Because the first and second allocated groups are inevitably separated in time, the period effect must be considered. Accordingly, this study performed a multilevel analysis to estimate the treatment effect that adjusted for a sequence and period effect.

There are some limitations among this study. Firstly, serum magnesium levels were not measured and monitored. Secondly, this study assigned participants to rate their pain intensity as pain at the moment of evaluation, which could be masked by recent intake of analgesic. Thirdly, the optimal regimen and magnesium pharmaceutical form for pain management remain unclear. Further investigation should implement this intervention on the patients who need to remove simultaneously maxillary third molar in the same surgery or evaluate the efficacy of other pharmaceutical forms of magnesium in the MTM surgery. Lastly, this was a preliminary study, and further studies with a larger sample size are necessary to determine a precise effect size.

As the result of this study, young healthy patients (both sexes), presented with impacted MTM requiring bone removal or/and tooth sectioning under local anesthesia, would benefit from using oral magnesium as adjuvant analgesic of ibuprofen. This intervention can also be implemented in surgery which has operative time less than 30 min approximately.

In conclusion, this randomized clinical trial suggests that the addition of the oral magnesium supplement (as magnesium oxide, citrate, and succinate) significantly alleviate pain at rest at 24 h, postoperatively, but this result might not provide clinically significant improvement in overall pain reduction, when combined with ibuprofen, following the MTM surgery.

## Figures and Tables

**Table 1:** Baseline demographic and characteristics by sequence and by total.

	Treatment sequence		Total (*n* = 50)
	Mg–placebo (*n* = 26)	Placebo–Mg (*n* = 24)	
Age, mean ± SD, year	20.07 ± 1.41	21.25 ± 2.25	20.64 ± 1.94
Female, *n* (%)	12 (46.15)	14 (58.33)	26 (52.0)
MDAS score (mean ± SD)	11.69 ± 3.08	10.25 ± 3.39	11 ± 3.2
Low level (< 15), *n* (%)	24 (92.31)	22 (91.67)	46 (92.00)
High level (≥ 15), *n* (%)	2 (7.69)	2 (8.33)	4 (8.00)
PCS score (mean ± SD)	17.15 ± 8.14	14.83 ± 9.50	16.04 ± 8.80
Low level (< 30), *n* (%)	24 (92.31)	24 (100.00)	48 (96.00)
High level (≥ 30), *n* (%)	2 (7.69)	0 (0)	2 (4.00)
Operation time, mean ± SD, minute	26.38 ± 9.53	29.58 ± 6.68	27.92 ± 8.36
Tooth status, *n* (%)			
Unerupted	15 (57.69)	16 (66.67)	31 (62.00)
Partial erupted	11 (43.31)	8 (33.33)	19 (38.00)
Tooth angulation, *n* (%)			
Mesioangular	20 (76.92)	11 (45.83)	31 (62.00)
Horizontal	6 (23.08)	13 (54.17)	19 (38.00)
Depth, *n* (%)			
Class I	3 (11.54)	3 (12.50)	6 (12.00)
Class II	20 (76.92)	18 (75.00)	38 (76.00)
Class III	3 (1.54)	3 (12.50)	6 (12.00)
Position, *n* (%)			
Position A	13 (50.00)	15 (62.50)	28 (56.00)
Position B	13 (50.00)	9 (37.50)	22 (44.00)

Abbreviations: MDAS, modified dental anxiety scale; Mg, magnesium; PCS, pain catastrophizing scale; SD, standard deviation.

**Table 2:** Primary outcome within 72 h following surgery analyzed in an intention-to-treat fashion.

Primary endpoints	Magnesium group (*n* = 25)	Placebo group (*n* = 25)	*p* value	Mean difference ^δ^ (95% CI)
Pain intensity at rest (HP-VAS score)				
6 h	54.60 ± 29.73	57.56 ± 38.90	0.764	−2.80 (−19.22–13.61)
24 h	30.80 ± 29.95	46.04 ± 31.19	0.044⁣^∗^	−15.08 (−29.01 to −1.14)
48 h	22.36 ± 25.34	33.24 ± 26.40	0.083	−10.72 (−22.08–0.63)
72 h	17.68 ± 26.87	22.12 ± 26.62	0.343	−4.28 (−14.50–5.93)
Pain intensity at movement (HP-VAS score)				
6 h	79.24 ± 35.89	75.32 ± 37.91	0.840	2.33 (−14.46–19.13)
24 h	53.24 ± 37.49	56.88 ± 39.54	0.388	−8.66 (−24.20–6.86)
48 h	40.40 ± 34.96	48.84 ± 38.42	0.204	−14.10 (−28.25–0.03)
72 h	26.04 ± 29.29	29.88 ± 24.79	0.292	−4.22 (−17.43–8.97)

*Note:* All data are presented as mean and standard deviations.

Abbreviations: CI, confidence interval; HP-VAS, Heft–Parker visual analog scale.

^*δ*^ Mean difference (magnesium–placebo) was analyzed using the multilevel linear mixed-effects modeling, adjusted for period effect and sequence effect.

⁣^∗^*p* values significant at the < 0.05 level.

**Table 3:** Secondary endpoints of the study.

	Magnesium group (*n* = 25)	Placebo group (*n* = 25)	*p* value
Number of cases who consumed rescue analgesic drug (*n*, %)	10 (40.00)	14 (56.00)	0.258^a^
Number of rescue analgesic drugs consumed (tablet, mean ± SD)	1.00 ± 2.10	1.68 ± 2.58	0.232^b^
Time to first rescue analgesic consumption (days, mean ± SD)	2.02 ± 1.35	1.65 ± 1.32	0.213^b^
Pain intensity at the time of first rescue analgesic consumption (HP-VAS, mean ± SD)	103.89 ± 26.63	109.67 ± 44.09	0.738^a^
Total number of cases who experienced adverse events from oral magnesium (*n*, %)	0 (0)		

Abbreviations: SD, standard deviation; HP-VAS, Heft–Parker visual analog scale.

^a^Data were analyzed with the signed-rank test.

^b^Data were analyzed with the chi-squared test.

⁣^∗^*p* values significant at the < 0.05 level.

**Table 4:** CONSORT 2010 checklist of information to include when reporting a randomized trial.

Section/topic	Item no.	Checklist item	Reported on page no.
Title and abstract			
	1a	Identification as a randomized trial in the title	1 and 2
	1b	Structured summary of trial design, methods, results, and conclusions (for specific guidance see CONSORT for abstracts)	1 and 2
Introduction			
Background and objectives	2a	Scientific background and explanation of rationale	3
	2b	Specific objectives or hypotheses	3
Methods			
Trial design	3a	Description of the trial design (such as parallel and factorial) including the allocation ratio	4
	3b	Important changes to methods after trial commencement (such as eligibility criteria), with reasons	N/A
Participants	4a	Eligibility criteria for participants	4
	4b	Settings and locations where the data were collected	4
Interventions	5	The interventions for each group with sufficient details to allow replication, including how and when they were actually administered	4–5
Outcomes	6a	Completely defined prespecified primary and secondary outcome measures, including how and when they were assessed	5–6
	6b	Any changes to trial outcomes after the trial commenced, with reasons	N/A
Sample size	7a	How sample size was determined	6
	7b	When applicable, explanation of any interim analyses and stopping guidelines	N/A
Randomization:			
Sequence generation	8a	Methods used to generate the random allocation sequence	4
	8b	Type of randomization; details of any restriction (such as blocking and block size)	4
Allocation concealment mechanism	9	Mechanism used to implement the random allocation sequence (such as sequentially numbered containers), describing any steps taken to conceal the sequence until interventions were assigned	4
Implementation	10	Who generated the random allocation sequence, who enrolled participants, and who assigned participants to interventions	4
Blinding	11a	If done, who was blinded after assignment to interventions (for example, participants, care providers, and those assessing outcomes) and how	4
	11b	If relevant, description of the similarity of interventions	4
Statistical methods	12a	Statistical methods used to compare groups for primary and secondary outcomes	6
	12b	Methods for additional analyses, such as subgroup analyses and adjusted analyses	6
Results			
Participant flow (a diagram is strongly recommended)	13a	For each group, the numbers of participants, who were randomly assigned, received intended treatment and were analyzed for the primary outcome	6–7
	13b	For each group, losses and exclusions after randomization, together with reasons	6–7
Recruitment	14a	Dates defining the periods of recruitment and follow-up	6–7
	14b	Why the trial ended or was stopped	N/A
Baseline data	15	A table showing baseline demographic and clinical characteristics for each group	17
Numbers analyzed	16	For each group, number of participants (denominator) included in each analysis and whether the analysis was by original assigned groups	6–7
Outcomes and estimation	17a	For each primary and secondary outcome, results for each group, and the estimated effect size and its precision (such as 95% confidence interval)	6–7, 15–16
	17b	For binary outcomes, presentation of both absolute and relative effect sizes is recommended	N/A
Ancillary analyses	18	Results of any other analyses performed, including subgroup analyses and adjusted analyses, distinguishing prespecified from exploratory	6–7,18
Harms	19	All important harms or unintended effects in each group (for specific guidance see CONSORT for harms)	7
Discussion			
Limitations	20	Trial limitations, addressing sources of potential bias, imprecision, and, if relevant, multiplicity of analyses	7–10
Generalizability	21	Generalizability (external validity and applicability) of the trial findings	10
Interpretation	22	Interpretation consistent with results, balancing benefits and harms, and considering other relevant evidence	7–10
Other information			
Registration	23	Registration number and name of the trial registry	4
Protocol	24	Where the full trial protocol can be accessed, if available	4
Funding	25	Sources of funding and other support (such as supply of drugs) and role of funders	11

## Data Availability

The data that support the findings of this study are available on request from the corresponding author. The data are not publicly available due to privacy or ethical restrictions.
